# Supplementary material for: Elucidating Flavonoid and Antioxidant Activity in Edible and Medicinal Herbs Woodwardia japonica (L.f.) Sm. Based on HPLC-ESI-TOF-MS and Artificial Neural Network Model: Response to Climatic Factors
Source: Molecules. 2023 Feb 20;28(4):1985. doi: 10.3390/molecules28041985 (PMC9964229; doi:10.3390/molecules28041985)
Supplement: Supplementary file 1 [file molecules-28-01985-s001.zip › molecules-2235194-supplementary.pdf]

Table S1 The Climate Factors of *Woodwardia japonica* Collected from Nine Main Production Areas

No.	Sample Sites	Habitat Soil Matrix	Average Temperature(℃)[a]	Maximum Temperature(℃)[a]	Minimum Temperature(℃)[a]	Average Precipitation(mm)[a]	Daytime Precipitation(mm)[a]	Overnight precipitation(mm)[a]	Sunshine duration(h)[a]	Average relative Humidity(%)[a]
S1	Wuyi Mountain (Jiangxi Province)	Soil	31.21	45.80	24.08	213.65	213.20	426.85	5.80	81.77
S2	Qingyuan County (Zhejiang Province)	Soil	31.14	45.00	24.48	108.70	108.28	6.01	5.43	81.97
S3	Wuyuan County (Jiangxi Province)	Soil	32.39	48.70	24.59	319.79	214.35	323.17	5.11	79.45
S4	Tianmu Mountain (Zhejiang Province)	Soil	31.15	47.07	24.00	108.49	214.63	112.15	6.49	87.39
S5	Huang Mountain (Anhui Province)	Sand	28.61	41.11	22.94	0.71	213.77	109.00	6.40	76.68
S6	Longquan County (Zhejiang Province)	Soil	31.19	44.15	24.68	214.54	108.34	322.88	5.07	74.58
S7	Fengyang Mountain (Zhejiang Province)	Dinas	31.72	47.97	24.15	59.94	23.94	83.87	5.90	79.19
S8	Jianyang County (Fujian Province)	Humus	31.62	47.76	24.45	319.36	318.86	321.77	5.66	81.35
S9	Wuyi Mountain (Fujian Province)	Soil	31.52	48.81	23.90	109.11	214.73	7.38	4.36	78.65

[a]Monthly mean value

Table S2. Pearson Correlation Coefficients Between TFC, Antioxidant Activity of *W. japonica* and Climate Factors [a]

Dependent variable/Independent variable	AT	MAT	MIT	AP	DP	OP	SD	ARH
Total flavonoid content	0.139	0.258	-0.092	0.206	0.317	0.208	-0.302	-0.049
O^{2-} radical scavenging activity	-0.007	0.135	-0.196	-0.055	-0.098	0.051	-0.066	-0.442
The reducing force on Fe^{3+}	0.083	0.238	-0.172	0.064	0.147	0.17	-0.057	-0.065
DPPH radical scavenging activity	-0.230	-0.151	-0.361	-0.521	-0.319	-0.276	0.181	0.151
ABTS radical scavenging activity	0.436	0.463	0.165	-0.033	-0.444	0.032	0.213	0.246

[a] $|r|\geq0.8$: extremely high correlation; $0.6\leq|r|<0.8$: high correlation; $0.4\leq|r|<0.6$: medium correlation; $0.2\leq|r|<0.4$: low correlation; $|r|<0.2$ no correlation

Table S3. Pearson Correlation Coefficients between Flavonoid Type of *W. japonica* and Climate Factors [a]

	AT	MAT	MIT	AP	DP	OP	SD	ARH
Compound 1	0.146	-0.195	0.456	0.454	0.045	0.392	-0.569	-0.343
Compound 2	-0.137	-0.028	-0.409	-0.149	0.083	0.248	0.332	0.231
Compound 3	-0.160	-0.135	-0.286	0.002	0.239	0.377	0.428	0.389
Compound 4	-0.274	0.022	-0.640	-0.521	-0.027	-0.228	0.249	0.100

Compound 5	-0.158	0.011	-0.453	-0.205	0.077	0.038	0.104	-0.015
Compound 6	0.097	0.394	-0.299	-0.276	0.083	-0.401	-0.425	-0.011
Compound 7	-0.005	0.033	0.011	-0.130	0.048	-0.098	0.423	0.582
Compound 8	0.026	0.290	-0.231	-0.401	0.011	-0.505	0.017	0.391

[a] $|r|\geq 0.8$: extremely high correlation; $0.6\leq|r|<0.8$: high correlation; $0.4\leq|r|<0.6$: medium correlation; $0.2\leq|r|<0.4$: low correlation; $|r|<0.2$ no correlation.

Compound 1-8: isotrifolin, rutin, myricetin deoxyhexoside, quercetin-3-rutinoside, luteolin, quercitrin, genestein G2, luteolin-4'-O-(6''-trans-caffeoyl) -z-d-glucopyranoside.
